# Scalable method for exploring phylogenetic placement uncertainty with custom visualizations using *treeio* and *ggtree*

**DOI:** 10.1002/imt2.269

**Published:** 2025-01-12

**Authors:** Meijun Chen, Xiao Luo, Shuangbin Xu, Lin Li, Junrui Li, Zijing Xie, Qianwen Wang, Yufan Liao, Bingdong Liu, Wenquan Liang, Ke Mo, Qiong Song, Xia Chen, Tommy Tsan‐Yuk Lam, Guangchuang Yu

**Affiliations:** ^1^ Department of Bioinformatics, School of Basic Medical Sciences Southern Medical University Guangzhou China; ^2^ Department of Cell Biology, School of Basic Medical Sciences Southern Medical University Guangzhou China; ^3^ State Key Laboratory of Applied Microbiology Southern China, Guangdong Provincial Key Laboratory of Microbial Culture Collection and Application, Guangdong Open Laboratory of Applied Microbiology, Guangdong Institute of Microbiology Guangdong Academy of Sciences Guangzhou China; ^4^ Department of General Surgery, Zhujiang Hospital Southern Medical University Guangzhou China; ^5^ Bioinformation Center of BioInforCloud, YuanDong International Academy of Life Sciences Hong Kong SAR China; ^6^ Central Laboratory of the Medical Research Center The First Affiliated Hospital of Ningbo University Ningbo China; ^7^ Department of Obstetrics and Gynecology The First Affiliated Hospital of Ningbo University Ningbo China; ^8^ State Key Laboratory of Emerging Infectious Diseases, School of Public Health The University of Hong Kong Hong Kong SAR China; ^9^ Laboratory of Data Discovery for Health Limited, 19W Hong Kong Science & Technology Parks Hong Kong SAR China

**Keywords:** *ggtree*, phylogenetic placement, placement uncertainty, *treeio*, visualization

## Abstract

In metabarcoding research, such as taxon identification, phylogenetic placement plays a critical role. However, many existing phylogenetic placement methods lack comprehensive features for downstream analysis and visualization. Visualization tools often ignore placement uncertainty, making it difficult to explore and interpret placement data effectively. To overcome these limitations, we introduce a scalable approach using *treeio* and *ggtree* for parsing and visualizing phylogenetic placement data. The *treeio*‐*ggtree* method supports placement filtration, uncertainty exploration, and customized visualization. It enhances scalability for large analyses by enabling users to extract subtrees from the full reference tree, focusing on specific samples within a clade. Additionally, this approach provides a clearer representation of phylogenetic placement uncertainty by visualizing associated placement information on the final placement tree.

## Highlights

- The *treeio* and *ggtree* packages significantly improve the comprehensive parsing and visualization of phylogenetic placement data.
- Utilizing *treeio* and *ggtree*, researchers can perform a range of sophisticated analysis tasks, including filtering specific phylogenetic placements and assessing the confidence levels of different placement positions.
- The advanced features of these tools enhance the understanding of evolutionary connections within metagenomic datasets.

## Graphical abstract

The **treeio** and **ggtree** packages provide robust tools for the comprehensive parsing and visualization of phylogenetic placement data. These tools enable detailed analyses, including placement filtration and uncertainty assessment, thereby facilitating deeper insights into evolutionary relationships within metagenomic datasets.

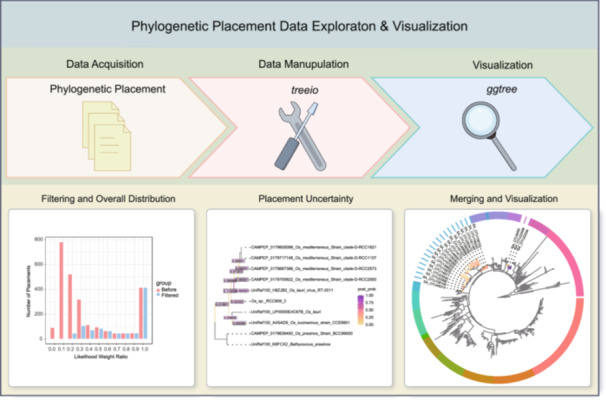

## INTRODUCTION

Phylogenetic analysis plays a crucial role in elucidating evolutionary relationships among species or genes by utilizing molecular sequence data. However, constructing and visualizing large phylogenetic trees is becoming increasingly challenging, particularly with expansive datasets derived from metagenomics sequencing and the rapidly growing collection of severe acute respiratory syndrome coronavirus 2 (SARS‐CoV‐2) genome sequences during the pandemic [1, 2]. Phylogenetic placement provides a practical solution for building extensive trees and identifying taxa by comparing the mutations of query sequences against the tips and nodes of a reference tree. Moreover, metagenomic (or metabarcoding) data, which typically contain a range of microbial taxa, require the characterization of operational taxonomic units (OTUs) through comparisons with reference sequences from known species. A primary task in metagenomics is taxon assignment, wherein organisms present in a sequenced sample are classified. Pairwise alignment and phylogenetic placement represent two alternative methods for taxonomic assignments [3]. While sequence alignment can be time‐intensive and lacks evolutionary context, phylogenetic placement is highly effective for taxon identification as it determines the evolutionary position of sequences in relation to a reference phylogenetic tree [4]. Consequently, phylogenetic placement methods are increasingly employed in genomic and metagenomic research [5]. Rather than reconstructing an entire evolutionary tree, phylogenetic placement incorporates new samples into an existing reference tree, saving both computational resources and time. By leveraging diverse reference data, enhancing sequence inference, and establishing evolutionary relationships, phylogenetic placement can yield accurate and insightful results. As applications of this method continue to expand, post‐analysis and visualization are becoming increasingly important for comprehensive interpretation and communication of findings.

Placement methods can be categorized into three types based on their distinct algorithmic approaches: Maximum Likelihood Placement, Ancestral‐Reconstruction‐Based Placement, and Distance‐Based Placement [6]. The jplace format, introduced in 2012, serves as a standard for storing phylogenetic placement data [7]. This JSON‐based format includes five key components: tree, fields, placements, metadata, and version. Various phylogenetic placement programs, such as *pplacer*, Evolutionary Placement Algorithm (*EPA*), *RAPPAS*, and our in‐house developed package *TIPars*, utilize this format [8, 9, 10, 11]. However, these tools generally lack integrated visualization capabilities for placement results. Visualization of phylogenetic placement data, therefore, relies on external jplace‐compatible programs, such as *iTOL*, *BoSSA* (https://CRAN.R-project.org/package=BoSSA), *guppy*, *Genesis*, and *Gappa* [12, 13, 14]. Some tools, like *iTOL*, offer straightforward visualization of placement data but lack features for in‐depth data exploration. Beyond tools based on phylogenetic tree placement, there exists a phylogenetic network placement tool, NetPlacer, which operates independently of tree‐like reference phylogeny [5]. As this tool diverges from tree‐based placement, it will not be discussed further in this article. For clarity, all subsequent references to “phylogenetic placement” in this article will pertain exclusively to “phylogenetic tree placement.”

Placement data have been applied in various analytical contexts, such as phylofactorization and diversity calculations, supported by tools like *scrapp* and *Genesis* [14, 15]. Additionally, specialized tools, such as *UShER* and *Cydrasil 3*, have been developed to support phylogenetic placement applications in specific research domains, including SARS‐CoV‐2 pandemic studies and cyanobacterial research, respectively [16, 17]. These tools offer curated reference resources but limited support for visual exploration. Unfortunately, most tools provide limited access to and visualization of phylogenetic placement uncertainty [18]. Different phylogenetic placement algorithms yield distinct placement criteria [6]. For example, *TIPars*, which employs a parsimony criterion, incorporates rules to identify the optimal placement among multiple possibilities [11]. To mitigate ambiguity, it is essential to support diverse data filtering methods. *BoSSA*, for instance, enables placement extraction into a table format that can be filtered to identify optimal placements based on various criteria, though it lacks sufficient visualization capabilities. Existing tools still do not fully address the need for filtering multiple placements in metabarcoding analyses, nor do they sufficiently support the exploration of placement uncertainty and customized visualization.

Visualizing placement data can vary based on analytical objectives [6]. The first approach involves generating the final tree in Newick format, which includes the inserted query sequence branches and can be visualized using standard tree‐viewing tools. However, this approach typically relies on the most likely placement and omits uncertain information. The second approach overlays all query sequences onto the reference tree, displaying placement counts to indicate whether query sequences cluster in specific tree regions. Additionally, for each query sequence, multiple placement locations with different likelihood weight ratios (LWRs) can be computed using maximum likelihood placement tools. Visualizing the distribution of LWRs across the reference tree can provide insight into placement uncertainty. For queries with placement in multiple locations, it is crucial to apply quality control to the placement results. Uncertainty metrics include not only LWR but also other algorithm‐specific criteria, such as the posterior probability calculated by *pplacer*.

To facilitate tree data management and visualization in R, we have developed a suite of packages, including *ggtree*, *treeio*, *tidytree*, and *ggtreeExtra* [19, 20, 21, 22, 23]. These packages support the analysis and visualization of phylogenetic placement data. In this work, we demonstrate methods for parsing and visualizing phylogenetic placement data using *treeio* and *ggtree*, enhancing their functionality to address current challenges and improve placement visualization effectiveness [19, 21].

Furthermore, we introduce a set of packages offering streamlined data manipulation and advanced visualization, facilitating alternative multiple placement filtering techniques within R. Unlike *BoSSA*, these packages leverage *ggplot2*'s graphics grammar, providing an elegant and flexible approach to data visualization [24]. With *treeio* and *tidytree*'s capabilities for data parsing, manipulation, and integration, metadata, and other associated data can be seamlessly incorporated into phylogenetic placement analyses. Additionally, *ggtree* and *ggtreExtra* enable customized visualizations to explore placement distributions and uncertainties effectively.

## RESULTS

### Evaluation of random access memory utilization

**Figure 1:**
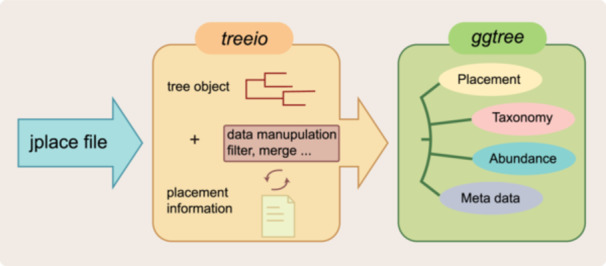
Workflow diagram illustrating the *treeio* and *ggtree* process for handling and visualizing phylogenetic placement data.

We introduced a framework designed to parse and explore phylogenetic placement data by leveraging the capabilities of the *treeio* and *ggtree* packages (Figure 1). To evaluate the efficiency of our packages in processing simulated tree data, we examined *treeio*'s performance in handling jplace files. As shown in Supporting Information S1: Figure S1 and Table S1, *treeio* exhibits high efficiency and avoids large random access memory (RAM) usage when reading jplace files. The computational resources are primarily determined by the number of rows for placement.

### Optimized placement filtering after parsing jplace files

Depicting the distribution and frequency of phylogenetic placements with associated uncertainty is challenging, especially as the number of queries grows. An effective strategy is to filter placements based on uncertainty metrics before downstream visualization. However, the absence of a standardized filtering method makes it essential for tools to provide filtering options based on diverse criteria.

**Figure 2:**
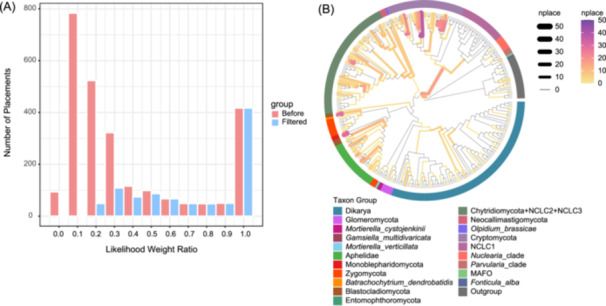
Visualization of placement data and likelihood weight ratio (LWR) distribution for *Holomycota* operational taxonomic units (OTUs) in the V4 region. (A) Histogram of LWR values before and after filtering. (B) Reference tree with filtered placements, where branch colors represent the number of placements. “Nplace” denotes the number of placements per branch. Gray branches indicate regions with no placements.

Here, we demonstrated placement filtering using the *Holomycota* V4 OTU data set [25]. In Figure 2A, approximately 65% of placements were discarded by applying a criterion to retain only those with the highest LWR values. Following filtering, placements are mapped onto the reference tree using *ggtree* (Figure 2B), where the Cryptomycota group emerged as the most abundant in the V4 region. Extracting placement data from jplace files enables users to remove low‐quality placements based on criteria such as LWR or posterior probability, then visualize results with custom annotations (e.g., color, numeric labels, line thickness, or symbols).

### Tool for exploring placement uncertainty of individual sequences

Within the *treeio*‐*ggtree* framework, we introduced utilities for exploring placement uncertainty, allowing users to investigate the distribution of LWR values across the reference tree for individual sequence placement. When reference trees are large, visualizing placement data can be challenging. In such cases, collapsing clades or extracting a subtree of interest can clarify placement patterns, particularly for sequences located on adjacent branches within specific clades.

**Figure 3:**
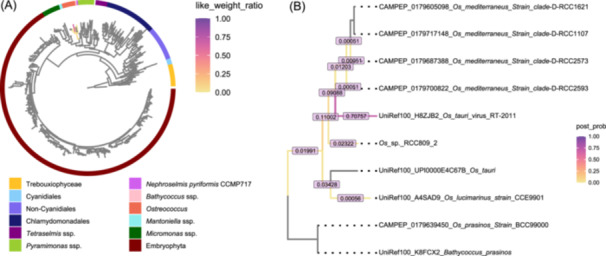
Visualization of LWR distribution for a single sequence, with a focus on an extracted subtree from a larger reference tree. (A) Full reference tree showing multiple placements of the sequence “*saltern1*” within a specific clade. (B) Detailed view of the *Ostreococcus* clade, where branch colors indicate posterior probability for *saltern1* placements. Branches are numerically labeled with posterior probability values. “post_prob” denotes posterior probability; “Os” refers to *Ostreococcus*.

As an example, we used the *Ostreococcus* data set [26], where an environmental sequence, named “saltern1,” with multiple placements and associated uncertainty values, was visualized on the reference tree. In Figure 3A, the reference tree appears compressed due to its many branches, while highlighted *Ostreococcus* clade displays branches colored by LWR values. Placement distribution across the clade aligns with findings from the original study, where the highest LWR value (darkest purple color, UniRef100_HBZJB2_*Os_tanu*_virus_RT‐2011) marks the optimal position for the query sequence.

These results underscore the utility of our approach in investigating placement uncertainty. In Figure 3B, an extracted *Ostreococcus* subtree provides a clearer structure with more space for annotations. Branch colors and numeric labels represent posterior probabilities, calculated using *pplacer*'s posterior probability mode. This example demonstrates the scalability of our method for handling large placements and enabling customized visual exploration.

### Merging placement information into the placement tree

Placement trees generated with placement branches can be saved in Newick format, though this format lacks essential placement information, such as LWR. For instance, IQ‐TREE2 adds new sequences to the reference tree by retaining only the best placement and outputs the result in Newick format, discarding other placement data. When multiple queries are placed on the same branches, corresponding placement details may not be visible on the backbone tree.

**Figure 4:**
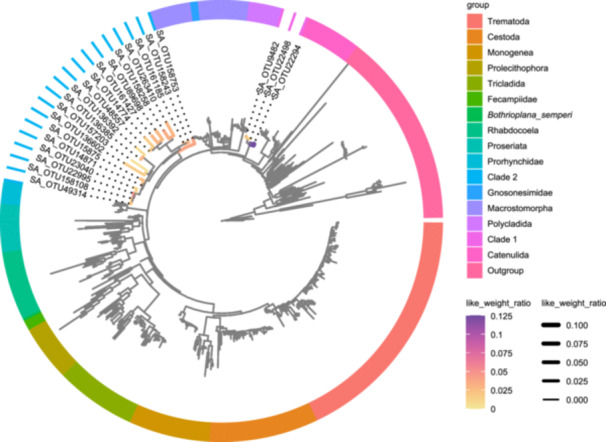
Maximum‐likelihood placement tree inferred from 22 OTUs with interesting placements and 455 reference sequences. Branch colors correspond to LWR values, with two clades, representing novel molecular lineages within Platyhelminthes, highlighted.

To address this issue, the *treeio*‐*ggtree* method allows users to merge placement information from a jplace file into the final placement tree in Newick format. In our example, *treeio* and *ggtree* integrate LWR values from a *jplace* file into a maximum‐likelihood placement tree. Using a data set previously analyzed in 2019 [27], we visualized LWR values across branches representing 22 OTUs, enhancing the interpretation of phylogenetic placement uncertainty (Figure 4). This approach offers a flexible solution for visualizing placement data.

## DISCUSSION

Placement visualization has become increasingly important with the availability of extensive reference phylogenetic tree resources from public datasets. However, existing tools often fail to support critical needs such as placement filtration, uncertainty exploration, and customized visualization. To bridge these gaps, we developed the *treeio*‐*ggtree* method, which enables comprehensive parsing and visualization of phylogenetic placement data, supporting placement filtration and uncertainty exploration.

We demonstrated the effectiveness of this method using three datasets, showcasing placement uncertainty visualization capabilities. The *ggtree* package provides highly customizable visualization options, supporting various annotation patterns. Additionally, this method offers practical solutions for visualizing large reference tree, making *treeio* and *ggtree* well‐suited for advanced phylogenetic placement analysis.

Our method is centered on enhancing data exploration and visualization; thus, no placement algorithm is integrated within our packages. Users are required to perform phylogenetic placement through alternative methods before utilizing our tools for data exploration and presentation. This method is compatible with both established and newly developed algorithms for phylogenetic placement. Furthermore, as our method is based on the R programming language, it may pose challenges for users unfamiliar with R. Future versions may include support for a Shiny web application to improve accessibility.

## CONCLUSION

We highlighted *ggtree*'s customizable visualization of placement uncertainty and annotation patterns using three datasets. This approach provides practical solutions for exploring phylogenetic placement.

## METHODS

### Software

In this work, we present a framework for parsing and exploring phylogenetic placement data using the *treeio* and *ggtree* packages. The *treeio* package enables users to parse phylogenetic trees with associated data from various file formats. For instance, jplace files can be parsed using *read.jplace()*, which captures placement counts. The resulting tree object retains associated placement data, which can then be visualized with *ggtree*. This framework allows users to access placement uncertainty data for downstream analysis and visualization on either the reference tree or the final placement tree. Additionally, users can apply custom quality control criteria to filter data, supporting further analyses. After filtering, placement counts can be summarized and mapped to the branches of the reference tree.

*Ggtree* supports diverse placement visualization techniques, offering customizable annotations. For an overview of placement distribution on the reference tree, placement counts can be mapped to the branch color and size. LWR, for example, can also be visualized to reflect phylogenetic placement uncertainty, aiding in the identification of optimal placements [6]. Tools like IQ‐TREE2 generate placement trees from placement results in Newick format but do not account for placement uncertainty; however, jplace‐extracted placement data can be integrated with Newick‐format placement trees.

### Data set collection

#### Simulated datasets

Simulated trees with varying tip counts were generated using the *ape* package's *rtree()* function and reformatted into jplace‐compatible text. Placement data were randomly generated without intrinsic meaning and used to assess performance in RAM.

#### Public datasets

To demonstrate the application of *treeio* and *ggtree* in phylogenetic placement analysis, we utilized three publicly available datasets (Supporting Information S1: Table S2). These datasets allowed us to evaluate filtration, uncertainty exploration, and visualization in phylogenetic placement.

### Evaluation of random access memory utilization

Simulated data were evaluated on Dell PowerEdge R750 with 2 CPUs (Intel(R) Xeon(R) Gold 6330 CPU @ 2.00 GHz), 112 threads, RAM: 503G. Results were tested by peakRAM (v 1.0.2) package. The test was run 10 times.

## AUTHOR CONTRIBUTIONS

**Meijun Chen**: Conceptualization; writing—original draft; investigation; software. **Xiao Luo**: Conceptualization; writing—review and editing; investigation. **Shuangbin Xu**: Conceptualization; writing—review and editing; investigation; software. **Lin Li**: Writing—review and editing; investigation. **Junrui Li**: Writing—review and editing; investigation. **Zijing Xie**: Writing—review and editing. **Qianwen Wang**: Writing—review and editing. **Yufan Liao**: Writing—review and editing. **Bingdong Liu**: Writing—review and editing. **Wenquan Liang**: Writing—review and editing. **Ke Mo**: Writing—review and editing. **Qiong Song**: Writing—review and editing. **Xia Chen**: Conceptualization; writing—review and editing. **Tommy Tsan‐Yuk Lam**: Conceptualization; writing—review and editing. **Guangchuang Yu**: Conceptualization; writing—original draft; writing—review and editing; software.

## CONFLICT OF INTEREST STATEMENT

The authors declare no conflicts of interest.

## ETHICS STATEMENT

No animals or humans were involved in this study.

## Supporting information


**Figure S1:** Evaluation of random access memory (RAM) utilization.

**Table S1:** Evaluation of random access memory (RAM) utilization using simulated data.
**Table S2:** Datasets used in this article.

## Data Availability

The data that support the findings of this study are openly available in Supplemental_ggtree_placement at https://github.com/YuLab-SMU/. The R script to generate figures is presented on GitHub: https://github.com/YuLab-SMU/Supplemental_ggtree_placement. Supplementary materials (figures, tables and graphical abstract) may be found in the online DOI or iMeta Science http://www.imeta.science/.

## References

[1] Li, Tianbao , Tao Huang , Cheng Guo , Ailan Wang , Xiaoli Shi , Xiaofei Mo , Qingqing Lu , et al. 2021. “Genomic Variation, Origin Tracing, and Vaccine Development of SARS‐COV‐2: A Systematic Review.” The Innovation 2: 100116. 10.1016/j.xinn.2021.100116 33997827 PMC8110321

[2] Lu, Guoqing , and Etsuko N. Moriyama . 2021. “2019nCoVR—A Comprehensive Genomic Resource for SARS‐COV‐2 Variant Surveillance.” The Innovation 2: 100150. 10.1016/j.xinn.2021.100150 34401863 PMC8357486

[3] Ewers, Isabelle , Lubomír Rajter , Lucas Czech , Frédéric Mahé , Alexandros Stamatakis , and Micah Dunthorn . 2023. “Interpreting Phylogenetic Placements for Taxonomic Assignment of Environmental DNA.” Journal of Eukaryotic Microbiology 70: e12990. 10.1111/jeu.12990 37448139

[4] Czech, Lucas , Pierre Barbera , and Alexandros Stamatakis . 2019. “Methods for Automatic Reference Trees and Multilevel Phylogenetic Placement.” Bioinformatics 35: 1151–1158. 10.1093/bioinformatics/bty767 30169747 PMC6449752

[5] Alamin, Md , and Kevin J. Liu . 2023. “Phylogenetic Placement of Aligned Genomes and Metagenomes With Non‐Tree‐Like Evolutionary Histories.” *Proceedings of the 14th ACM International Conference on Bioinformatics, Computational Biology, and Health Informatics, 1–10*. Houston, TX USA: ACM. 10.1109/TCBBIO.2024.3519311 40811323

[6] Czech, Lucas , Alexandros Stamatakis , Micah Dunthorn , and Pierre Barbera . 2022. “Metagenomic Analysis Using Phylogenetic Placement—A Review of the First Decade.” Frontiers in Bioinformatics 2: 871393. 10.3389/fbinf.2022.871393 36304302 PMC9580882

[7] Matsen, Frederick A. , Noah G. Hoffman , Aaron Gallagher , and Alexandros Stamatakis . 2012. “A Format for Phylogenetic Placements.” PLoS One 7: e31009. 10.1371/journal.pone.0031009 22383988 PMC3284489

[8] Berger, Simon A. , Denis Krompass , and Alexandros Stamatakis . 2011. “Performance, Accuracy, and Web Server for Evolutionary Placement of Short Sequence Reads under Maximum Likelihood.” Systematic Biology 60: 291–302. 10.1093/sysbio/syr010 21436105 PMC3078422

[9] Linard, Benjamin , Krister Swenson , and Fabio Pardi . 2019. “Rapid Alignment‐Free Phylogenetic Identification of Metagenomic Sequences.” Bioinformatics 35: 3303–3312. 10.1093/bioinformatics/btz068 30698645

[10] Matsen, Frederick A. , Robin B. Kodner , and E. Virginia Armbrust . 2010. “Pplacer: Linear Time Maximum‐Likelihood and Bayesian Phylogenetic Placement of Sequences Onto a Fixed Reference Tree.” BMC Bioinformatics 11: 538. 10.1186/1471-2105-11-538 21034504 PMC3098090

[11] Ye, Yongtao , Marcus H. Shum , Joseph L. Tsui , Guangchuang Yu , David K. Smith , Huachen Zhu , and Joseph T. Wu , et al. 2024. “Robust expansion of phylogeny for fast‐growing genome sequence data.” PLOS Computational Biology 20: e1011871. 10.1371/journal.pcbi.1011871 38330139 PMC10898724

[12] Letunic, Ivica , and Peer Bork . 2019. “Interactive Tree of Life (iTOL) v4: Recent Updates and New Developments.” Nucleic Acids Research 47: W256–W259. 10.1093/nar/gkz239 30931475 PMC6602468

[13] Matsen, Frederick A. , and Steven N. Evans . 2013. “Edge Principal Components and Squash Clustering: Using the Special Structure of Phylogenetic Placement Data for Sample Comparison.” PLoS One 8: e56859. 10.1371/journal.pone.0056859 23505415 PMC3594297

[14] Czech, Lucas , Pierre Barbera , and Alexandros Stamatakis . 2020. “Genesis and Gappa: Processing, Analyzing and Visualizing Phylogenetic (Placement) Data.” Bioinformatics 36: 3263–3265. 10.1093/bioinformatics/btaa070 32016344 PMC7214027

[15] Barbera, Pierre , Lucas Czech , Sarah Lutteropp , and Alexandros Stamatakis . 2021. “SCRAPP: A Tool to Assess the Diversity of Microbial Samples from Phylogenetic Placements.” Molecular Ecology Resources 21: 340–349. 10.1111/1755-0998.13255 32996237 PMC7756409

[16] Turakhia, Yatish , Bryan Thornlow , Angie S. Hinrichs , Nicola De Maio , Landen Gozashti , Robert Lanfear , David Haussler , and Russell Corbett‐Detig . 2021. “Ultrafast Sample Placement on Existing Trees (UShER) Enables Real‐Time Phylogenetics for the SARS‐COV‐2 Pandemic.” Nature Genetics 53: 809–816. 10.1038/s41588-021-00862-7 33972780 PMC9248294

[17] Roush, Daniel , Ana Giraldo‐Silva , and Ferran Garcia‐Pichel . 2021. “Cydrasil 3, A Curated 16S rRNA Gene Reference Package and Web App for Cyanobacterial Phylogenetic Placement.” Scientific Data 8: 230. 10.1038/s41597-021-01015-5 34475414 PMC8413452

[18] Theys, Kristof , Philippe Lemey , Anne‐Mieke Vandamme , and Guy Baele . 2019. “Advances in Visualization Tools for Phylogenomic and Phylodynamic Studies of Viral Diseases.” Frontiers in Public Health 7: 208. 10.3389/fpubh.2019.00208 31428595 PMC6688121

[19] Yu, Guangchuang , David K. Smith , Huachen Zhu , Yi Guan , and Tommy Tsan‐Yuk Lam . 2017. “Ggtree: An R Package for Visualization and Annotation of Phylogenetic Trees with Their Covariates and Other Associated Data.” Methods in Ecology and Evolution 8: 28–36. 10.1111/2041-210X.12628

[20] Yu, Guangchuang , Tommy Tsan‐Yuk Lam , Huachen Zhu , and Yi Guan . 2018. “Two Methods for Mapping and Visualizing Associated Data on Phylogeny Using Ggtree.” Molecular Biology and Evolution 35: 3041–3043. 10.1093/molbev/msy194 30351396 PMC6278858

[21] Wang, Li‐Gen , Tommy Tsan‐Yuk Lam , Shuangbin Xu , Zehan Dai , Lang Zhou , Tingze Feng , Pingfan Guo , et al. 2020. “Treeio: An R Package for Phylogenetic Tree Input and Output with Richly Annotated and Associated Data.” Molecular Biology and Evolution 37: 599–603. 10.1093/molbev/msz240 31633786 PMC6993851

[22] Xu, Shuangbin , Zehan Dai , Pingfan Guo , Xiaocong Fu , Shanshan Liu , Lang Zhou , Wenli Tang , et al. 2021. “GgtreeExtra: Compact Visualization of Richly Annotated Phylogenetic Data.” Molecular Biology and Evolution 38: 4039–4042. 10.1093/molbev/msab166 34097064 PMC8382893

[23] Yu, Guangchuang . 2022. Data Integration, Manipulation and Visualization of Phylogenetic Trees (1st Ed.). New York: Chapman and Hall/CRC.

[24] Wickham, Hadley . 2016. Ggplot2: Elegant Graphics for Data Analysis. Cham: Springer.

[25] Arroyo, Alicia S. , David López‐Escardó , Eunsoo Kim , Iñaki Ruiz‐Trillo , and Sebastián R. Najle . 2018. “Novel Diversity of Deeply Branching Holomycota and Unicellular Holozoans Revealed By Metabarcoding in Middle Paraná River, Argentina.” Frontiers in Ecology and Evolution 6: 99. 10.3389/fevo.2018.00099

[26] Monier, Adam , Aurélie Chambouvet , David S. Milner , Victoria Attah , Ramón Terrado , Connie Lovejoy , Hervé Moreau , et al. 2017. “Host‐Derived Viral Transporter Protein for Nitrogen Uptake in Infected Marine Phytoplankton.” Proceedings of the National Academy of Sciences 114: E7489. 10.1073/pnas.1708097114 PMC5594687 28827361

[27] Mitsi, Konstantina , Alicia S. Arroyo , and Iñaki Ruiz‐Trillo . 2019. “A Global Metabarcoding Analysis Expands Molecular Diversity of Platyhelminthes and Reveals Novel Early‐Branching Clades.” Biology Letters 15: 20190182. 10.1098/rsbl.2019.0182 31506037 PMC6769146

