# Supplementary material for: Scalable method for exploring phylogenetic placement uncertainty with custom visualizations using treeio and ggtree
Source: Imeta. 2025 Jan 12;4(1):e269. doi: 10.1002/imt2.269 (PMC11865327; doi:10.1002/imt2.269)
Supplement: Supplementary file 1 — Figure S1: Evaluation of random access memory (RAM) utilization. [file IMT2-4-e269-s002.docx]

**Supplementary materials to**

**Scalable method for exploring phylogenetic placement uncertainty with custom visualizations using *treeio* and *ggtree***

**Running title**: Exploring phylogenetic placement using *treeio* and *ggtree*

Meijun Chen^1,2#^, Xiao Luo^1#^, Shuangbin Xu^1#^, Lin Li^1^, Junrui Li^1^, Zijing Xie^1^, Qianwen Wang^1^, Yufan Liao^1^, Bingdong Liu^1,3^, Wenquan Liang^2^, Ke Mo^4,5^, Qiong Song^5^, Xia Chen^6,7*^, Tommy Tsan-Yuk Lam^8,9*^, Guangchuang Yu^1*^

^1^Department of Bioinformatics, School of Basic Medical Sciences, Southern Medical University, Guangzhou, 510515, China

^2^Department of Cell Biology, School of Basic Medical Sciences, Southern Medical University, Guangzhou, 510515, China

^3^State Key Laboratory of Applied Microbiology Southern China, Guangdong Provincial Key Laboratory of Microbial Culture Collection and Application, Guangdong Open Laboratory of Applied Microbiology, Guangdong Institute of Microbiology, Guangdong Academy of Sciences, Guangzhou, 510075, China

^4^Department of General Surgery, Zhujiang Hospital, Southern Medical University, Guangzhou, 510280, China

^5^Bioinformation Center of BioInforCloud, YuanDong International Academy of Life Sciences, Hong Kong SAR, 999077, China

^6^Central Laboratory of the Medical Research Center, The First Affiliated Hospital of Ningbo University, Ningbo, 315010, China

^7^Department of Obstetrics and Gynecology, The First Affiliated Hospital of Ningbo University, Ningbo, 315010, China

^8^State Key Laboratory of Emerging Infectious Diseases, School of Public Health, The University of Hong Kong, Hong Kong SAR, 999077, China

^9^Laboratory of Data Discovery for Health Limited, 19W Hong Kong Science & Technology Parks, Hong Kong SAR, 999077, China

^#^ These authors contributed equally: Meijun Chen, Xiao Luo, Shuangbin Xu.

^*^Correspondence: [gcyu1@smu.edu.cn](mailto:gcyu1@smu.edu.cn) (Guangchuang Yu), [ttylam@hku.hk](mailto:ttylam@hku.hk) (Tommy Tsan-Yuk Lam) , [chenx_fsyyy@163.com](mailto:chenx_fsyyy@163.com) (Xia Chen)

Figure S1 Evaluation of random access memory (RAM) Utilization. (A) Performance in elapsed time. (B) Performance in RAM usage. Num_Placement: Number of Rows for Placement. Num_TreeTips: Number of Tips for reference tree.
